# Efficacy and Safety of Subthreshold Micropulse Yellow Laser for Persistent Diabetic Macular Edema After Vitrectomy: A Pilot Study

**DOI:** 10.3389/fphar.2022.832448

**Published:** 2022-04-06

**Authors:** Vincenza Bonfiglio, Robert Rejdak, Katarzyna Nowomiejska, Sandrine Anne Zweifel, Maximilian Robert Justus Wiest, Giovanni Luca Romano, Claudio Bucolo, Lucia Gozzo, Niccolò Castellino, Clara Patane, Corrado Pizzo, Michele Reibaldi, Andrea Russo, Antonio Longo, Matteo Fallico, Iacopo Macchi, Maria Vadalà, Teresio Avitabile, Ciro Costagliola, Kamil Jonak, Mario Damiano Toro

**Affiliations:** ^1^ Department of Experimental Biomedicine and Clinical Neuroscience, Ophthalmology Section, University of Palermo, Palermo, Italy; ^2^ Chair and Department of General and Pediatric Ophthalmology, Medical University of Lublin, Lublin, Poland; ^3^ Department of Ophthalmology, University of Zurich, Zurich, Switzerland; ^4^ Department of Biomedical and Biotechnological Sciences, Section of Pharmacology, University of Catania, Catania, Italy; ^5^ Center for Research in Ocular Pharmacology—CERFO, University of Catania, Catania, Italy; ^6^ Department of Ophthalmology, University of Catania, Catania, Italy; ^7^ Department of Surgical Sciences, Eye Clinic Section, University of Turin, Turin, Italy; ^8^ Eye Clinic Department of Neuroscience, Reproductive and Odontostomatological Sciences, University of Naples Federico II, Naples, Italy; ^9^ Department of Clinical Neuropsychiatry, Medical University of Lublin, Lublin, Poland; ^10^ Department of Computer Science, Lublin University of Technology, Lublin, Poland; ^11^ Eye Clinic, Public Health Department, University of Naples Federico II, Naples, Italy

**Keywords:** subthreshold micropulse laser, tractional DME, OCT angiography, inflammation, diabetic retinopathy

## Abstract

**Aim:** To examine the effect of subthreshold micropulse yellow laser (SMYL) on best-corrected visual acuity (BCVA), central macular thickness (CMT), and optical coherence tomography angiography (OCT-A) changes in eyes with persistent diabetic macular edema (DME) after pars plana vitrectomy (PPV) for tractional DME (TDME).

**Patients and Methods:** In a comparative study, 95 eyes of 95 consecutive patients with persistent DME were prospectively enrolled. The SMYL group (54 eyes) was treated with SMYL 6 months after PPV, while the control group (41 eyes) was followed up without treatment. BCVA and CMT by OCT were analyzed at baseline and 3 and 6 months. Additionally, parameters such as the vessel density (VD) in the superficial capillary plexus (SCP) and deep capillary plexus (DCP), respectively, and the area of the foveal avascular zone (FAZ) were also evaluated on OCT-A.

**Results:** There were no significant differences between both groups in demographic data. In the SMYL group, mean BCVA was significantly increased [F(2,106) = 17.25; *p* < 0.001; $\eta_{p}^{2}$ = 0.246] from 51.54 ± 13.81 ETDRS letters at baseline to 57.81 ± 12.82 ETDRS letters at 3 months (*p* < 0.001) and 57.83 ± 13.95 EDTRS letters at 6 months (*p* < 0.001), respectively. In comparison to the control group, BCVA values were statistically significantly higher in the SMYL group at 3 and 6 months, respectively. Mean CMT significantly decreased [F(2,106) = 30.98; *p* < 0.001; $\eta_{p}^{2}$ = 0.368] from the baseline value 410.59 ± 129.91 μm to 323.50 ± 89.66 μm at 3 months (*p* < 0.001) and to 283.39 ± 73.45 μm at 6 months (*p* < 0.001). CMT values were significantly lower in the SMYL group (*p* < 0.001), especially at 6 months follow-up time (*p* < 0.001) compared with the control group. Parafoveal VD in the SCP and DCP was significantly higher in the SMYL group in comparison to the control group, respectively, at 3-month (SCP *p* < 0.001; DCP *p* < 0.001) and 6-month follow-up (SCP *p* < 0.001; DCP *p* < 0.001). FAZ area was also significantly smaller in the SMYL group at 6-month follow-up (*p* = 0.001). There were no adverse SMYL treatment effects.

**Conclusion:** SMYL therapy may be a safe and effective treatment option in eyes with persistent macular edema following PPV for TDME.

## Introduction

Diabetic macular edema (DME) is a major disorder with increasing public health importance across the world (Klein et al., 1995).

The pathogenesis of DME is complex and multifactorial, and it is the result of the disruption of the blood–retinal barrier (BRB) (Bandello et al., 2017; Ceravolo et al., 2020). A study by optical coherence tomography (OCT) has shown abnormalities in the vitreomacular interface (VMI) up to 75% of eyes with DME (Ophir et al., 2010). In particular, vitreomacular traction (VMT), reported from 4 to 25% (Thomas et al., 2005), is a relevant factor in the development and persistence of DME. Indeed, the retina interface can be distorted by attached vitreous, epiretinal membranes and abnormal taut vitreomacular adhesions (Hartley et al., 2008).

To date, intravitreal therapy (IVT) with anti-vascular endothelial growth factor (VEGF) agents or steroids is considered a first-line treatment of DME (Bucolo et al., 2018; Kodjikian et al., 2019; Arumuganathan et al., 2021; Elfalah et al., 2021). However, the treatment of tractional DME (TDME) with IVT of anti-VEGF or corticosteroids may be poorly effective due to a possible influence of tractional forces (Sadiq et al., 2016; Chang et al., 2017). In such cases, pars plana vitrectomy (PPV) has been proven to be an effective therapeutic option in the resolution of DME, removing the tractional cause that is involved in its pathogenesis (Flikier et al., 2019). Despite surgery, persistent or recurrent DME can occur, and it is difficult to treat due to the increased clearance of medications in the vitreous cavity of vitrectomized eyes (Gunay and Erdogan, 2021). Previous studies (Yanyali et al., 2007; Pessoa et al., 2018; Pessoa et al., 2019; Arumuganathan et al., 2021) observed the persistence of DME up to 22% of eyes with TDME treated with PPV.

Currently, no treatment algorithm exists for recurrent or persistent DME in vitrectomized eyes although the use of dexamethasone (DEX) (Reibaldi et al., 2012; Bonfiglio et al., 2017) or fluoroquinolone acetonide (FA) implants (Meireles et al., 2017; Pessoa et al., 2018) has been proved to be effective in these cases, playing an anti-inflammatory role, even if a risk of cataract progression, ocular hypertension, and endophthalmitis was reported (Bonfiglio et al., 2017; Bucolo et al., 2018; Pessoa et al., 2018). Laser photocoagulation has been historically represented as the main option for the treatment of DME. Subthreshold micropulse yellow laser (SMYL) is a new treatment option that turns out to be safe and effective in the treatment of macular edema induced by different retinal diseases, including DME in naïve eyes. SMYL uses a photo-stimulation process with repetitive short pulses at low temperatures through which the tissue is preserved (Frizziero et al., 2021). Yellow light has an excellent absorption rate for O2 Hb and is not absorbed by foveal pigments such as lutein and zeaxanthin, thus allowing central macular edema treatment without foveal damage (Gawecki 2019). This method is a revolutionary alternative when compared to a conventional continuous wavelength laser. Previous studies have demonstrated that, in eyes with naïve DME, SMYL treatment plays an anti-inflammatory effect, reducing the aqueous humor (AH) concentration of inflammatory cytokines secreted by retinal glial cells (GLCs), both Müller cells (MCs) and microglial cells (MGCs), and the number of hyper-reflective retinal spots (HRS) (Midena et al., 2019; Midena et al., 2020; Vujosevic et al., 2020).

This pilot study aimed to evaluate the functional and anatomical outcomes and the rate of side effects of SMYL for the treatment of persistent DME after PPV for TDME in comparison with a control group observed after PPV.

## Patients and Methods

In this perspective, comparative non-randomized pilot study, all consecutive pseudophakic patients with a persistent DME after PPV for TDME at the Retina Division of the Chair and Department of General and Pediatric Ophthalmology at the University of Lublin, Poland between March 2019 and September 2020 were evaluated. The study, compliant with the tenets of the Declaration of Helsinki, was approved by our Institutional Review Board (n° KE-0254/132/2019). Every patient signed written informed consent for the treatment of personal data.

In the present study, the included eyes with persistent DME after PPV were divided into two groups: DME eyes who received micropulse subthreshold laser treatment (SMYL group) and matched DME eyes observed after PPV without treatment (control group). All the eyes with persistent DME after PPV, included in the study, underwent a 25-gauge PPV, associated with epiretinal membrane (ERM) peeling and gas injection performed by the same surgeon (R.R.) under local anesthesia. If necessary, a posterior capsulotomy was performed at the beginning of PPV. The staining of the ERM was performed in all patients by brilliant blue G (BBG).

Persistent DME was defined as a persistent central macular thickness (CMT) ≥ 300 μm by spectral-domain (SD) OCT for at least 6 months after PPV, and no response to conventional treatments (steroid and non-steroidal anti-inflammatory eye drops, and tablets such as oral indomethacin) (Elkayal et al., 2021).

The inclusion criteria for eyes with persistent DME were: a confirmed diagnosis of diabetes mellitus Type 2 as defined by the World Health Organization (WHO) criteria; an age of ≥18 years; best-corrected visual acuity (BCVA) between 70 and 35 ETDRS letters; an absence of macular ischemia assessed with fluorescein angiography (FFA) and a follow-up of at least 6 months after SMYL laser treatment.

The exclusion criteria were co-existence of any eye disease that may affect the visual outcome including glaucoma, macular hole, age-related macular degeneration, vascular occlusion, axial length >26 mm, amblyopia, active proliferative diabetic retinopathy, and vitreous hemorrhage. The patients affected by chorioretinal atrophy in the macular area or lipid exudative disorders and grid or focal laser treatments, or IVT of any anti-VEGF agents or steroids during 6 months before PPV were also excluded. In the SMYL group, laser treatment with 577 nm SMYL photo-stimulation (IRIDEX IQ 577TM, IRIDEX, Mountain View, CA, United States) was performed on the macula by the same ophthalmologist (KN). The Area-Centralis lens (Volk Optical, Mentor, OH, United States) was used and the micropulse laser power was obtained for each eye after a continuous wave test burn that was located more than 3-disc diameters from the foveal center outside the vascular arcades in a non-edematous area. A 200 μm diameter spot was tested with a pulse duration of 200 msec and a power of 50 mW in a non-edematous area. The power was increased at 10 mW increments (whilst advancing the laser to non-edematous areas immediately beside the previous test site) until a barely visible tissue reaction (white color) was observed. The SMYL treatment was performed on the edema site, switching on a 5% duty cycle and adjusting the power to four times the test spot threshold. Two hundred milliseconds of exposure and 4 grids (7 × 7) with confluent spots of 200 μm (0.00 spacing), including the foveal center were used. The setting, including the spot size, lens, and duration remained the same as it was in the test spot (Verdina et al., 2020).

Retreatment was performed at 3 months from the first treatment, using the same power setting, if CMT was >300 µm, or the retinal thickness decrease in the treated ETDRS quadrant (on OCT map) was less than 20% of the baseline value (Vujosevic et al., 2020).

After SMYL treatment non-steroidal anti-inflammatory eye drops were administered twice a day for 1 month in all cases.

In both groups, functional and anatomical findings were recorded at baseline, 3 and 6 months, including autofluorescence.

A single, independent, well-trained, experienced ophthalmologist measured the BCVA using the Early Treatment Diabetic Retinopathy Study (ETDRS) charts at a 4 m distance. For statistical analysis, visual acuity was scored as the total number of letters read correctly (ETDRS score).

OCT angiography (OCT-A) was performed using an XR Avanti AngioVue OCT-A (version 2017.1.0.151AngioVue Phase 7 software with PAR) in the Angio Retina mode and a scanning area of 6 × 6 mm. The retinal vascular layers were visualized and segmented based on the default settings of the automated software algorithm embedded in the XR Avanti AngioVue OCT-A.

The three-dimensional projection artifacts removal (3D-PAR) algorithm was applied to simplify the OCT-A imaging interpretation by enhancing the depth resolution of vascular layers. This new algorithm retains the flow signal from real blood vessels, while suppressing the projected flow signal in deeper layers, avoiding downward tails on cross-sectional angiograms, and duplicated vascular patterns on *en* face angiograms (Iafe et al., 2016).

The images were reviewed by two retinal specialists for the correctness of segmentation; if segmentation errors were observed, they were corrected using the segmentation editing and propagation tool embedded in the AngioVue system.

The updated AngioVue software automatically calculates a single foveal avascular zone (FAZ) value as automated FAZ boundary detection provided by the AngioVue software, applied on a retinal slab that includes both superficial and deep vascular plex [from the internal limiting membrane (ILM) to outer plexus layer +10 µm]. This protocol was used based on the recent studies validating a single merged quantitative measurement of the FAZ (Coscas et al., 2016; Bonfiglio et al., 2019).

The vessel density (VD) was defined as the percentage area occupied by vessels in a circular region of interest (ROI) centered on the center of the FAZ with a diameter of 3 mm included inside the 6 × 6 mm scan area (Wiest et al., 2021). The AngioVue software automatically splits the ROI into three fields: the foveal area, a central circle with a diameter of 1 mm; and the parafoveal area and perifoveal area of 3.0 and 6.0 mm, respectively. The foveal and parafoveal density of superficial and deep capillary plex (SCP and DCP) were analyzed. Low-quality OCT-A images with signal strength index <50 were excluded from the analysis (Bonfiglio et al., 2019).

CMT was assessed by the same OCT system (version 2017.1.0.151 AngioVue Phase 7 software with PAR) at the same time as the retinal vasculature using the retinal map mode, which covered a 6 × 6 mm area centered at the fovea. CMT was automatically measured as the average macular thickness within a scope of 1 mm in diameter, centered around the fovea (Bonfiglio et al., 2019).

At the baseline examination, each radial SD-OCT scan and each OCT-A scan were marked as the patient’s baseline and it was used as a reference for the subsequent scans using the “follow-up” function, assuring that the scans would be performed in the same position. Two masked expert investigators interpreted the SD-OCT images. When there was disagreement, a third investigator was consulted for the final decision.

### Statistical Analysis

For statistical analysis, BCVA, CMT, and FAZ detected at baseline (6 months after PPV) and after SMYL laser treatment (3 and 6 months) were evaluated and presented as means ± standard deviations (SD) in both groups. The mixed model repeated measures analysis of variance (ANOVA) with *η*_p_^2^ as an effect size indicator was used to determine whether there were any significant differences between the baseline, 3 and 6 months of the follow-up. For post hoc comparison, Bonferroni test was used. A *p* value less than 0.003 was considered statistically significant (including correction for multiple comparisons). For statistical analysis of the data, the Statistical Package for the Social Sciences, v.17.0 for Windows (SPSS, Chicago, Ill., United States) has been applied.

## Results

In our study, 97 eyes of 97 patients with persistent DME after PPV for TDME met the inclusion criteria. Fifty-six eyes of 56 patients in the SMYL group were treated by SMYL (SMYL Group), while 41 eyes of 41 patients in the control group were observed. Two, out of 56 patients of the SMYL group, were excluded because lost at the 3 months follow-up. Therefore, 54 consecutive eyes of 54 patients were used for the data analysis.

Baseline demographics and clinical characteristics of both SMYL and control groups are shown in Table 1.

**TABLE 1:** Demographics and clinical characteristics of the SMYL and control group at baseline.

	SMYL group (*n* = 54 eyes)	Control group (*n* = 41 eyes)	*p* value
Mean age (years) ±SD	69.65 ± 11.30	67.81 ± 12.82	0.366
Gender (male/female ratio)	24/30	19/22	0.215
Mean duration of diabetes ±SD	13.67 ± 6.63	18.65 ± 3.72	0.291
Mean hemoglobin A1c level (%) ±SD	8.1 ± 2.1%	7.70 ± 0.81%	0.136

Abbreviations: SMYL, subthreshold micropulse yellow laser; SD, standard deviation.

No statistically significant differences were observed between both groups at the baseline.

In the SMYL Group, the post hoc comparison showed that mean BCVA increased significantly from 51.54 ± 13.81 ETDRS letters (baseline) to 57.81 ± 12.82 ETDRS letters (*p* < 0.001) at 3 months and 57.83 ± 13.95 (*p* < 0.001) ETDRS letters at 6 months, respectively. No statistically significant differences were found between 3 and 6 months (*p* = 1, Table 2).

**TABLE 2:** Comparison of mean BCVA, CMT, and OCT-A parameters over the SMYL group.

	Baseline	3 months	6 months	ANOVA	
				*p* value	$\eta_{p}^{2}$
Mean BCVA (ETDRS letters) ±SD	51.54 ± 13.81	57.81 ± 12.82	57.83 ± 13.95	< 0.001*	0.246
Mean CMT (µm) ±SD	410.59 ± 129.91	331.01 ± 89.66	283.39 ± 73.45	< 0.001^†^	0.368
Mean foveal VD SCP (%) ±SD	25.51 ± 5.96	25.41 ± 5.16	27.53 ± 4.97	0.055	0.054
Mean foveal VD DCP (%) ±SD	25.38 ± 7.15	25.76 ± 7.48	27.09 ± 4.98	0.373	0.018
Mean parafoveal VD SCP (%) ±SD	42.17 ± 3.42	42.50 ± 3.75	43.43 ± 4.48	0.136	0.036
Mean parafoveal VD DCP (%) ±SD	47.69 ± 3.39	48.91 ± 3.86	47.98 ± 3.44	0.227	0.027
Mean FAZ (mm^2^) ±SD	0.29 ± 0.09	0.29 ± 0.10	0.31 ± 0.12	0.478	0.014

*Post hoc baseline vs. 3 months: *p* < 0.001; baseline vs. 6 months: *p* < 0.001; 3 vs. 6 months: *p* = 1

^†^Post hoc baseline vs. 3 months: *p* < 0.001; baseline vs. 6 months: *p* < 0.001; 3 vs. 6 months: *p* = 0.012.

Abbreviations: BCVA, best-corrected visual acuity; CMT, central macular thickness; OCT-A, optical coherence tomography angiography; SMYL, subthreshold micropulse yellow laser; ANOVA, analysis of variance; $\eta_{p}^{2}$ partial etha squared; ETDRS, Early Treatment Diabetic Retinopathy Study; SD, standard deviation; µm, micrometers; VD, vessel density; SCP, superficial capillary plexus; DCP, deep capillary plexus; FAZ, foveal avascular zone; mm^2^, millimeter squared.

**FIGURE 1:**
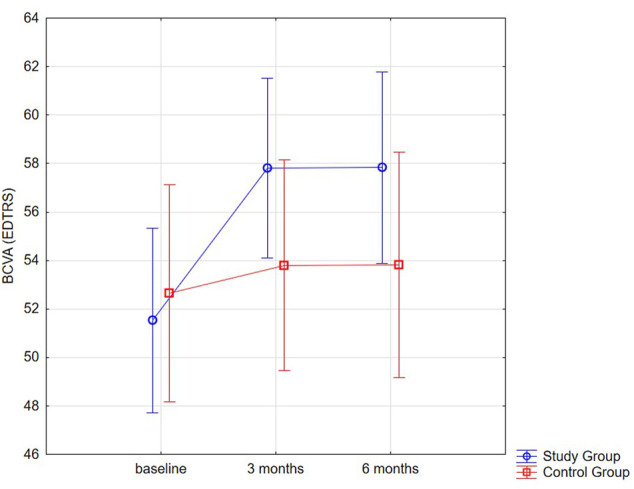
Distribution plot for BCVA (EDTRS letters) mean values in both groups at baseline and 3 and 6 month.

In comparison to the control group, BCVA values were statistically significantly higher in the SMYL group (Figure 1).

**FIGURE 2:**
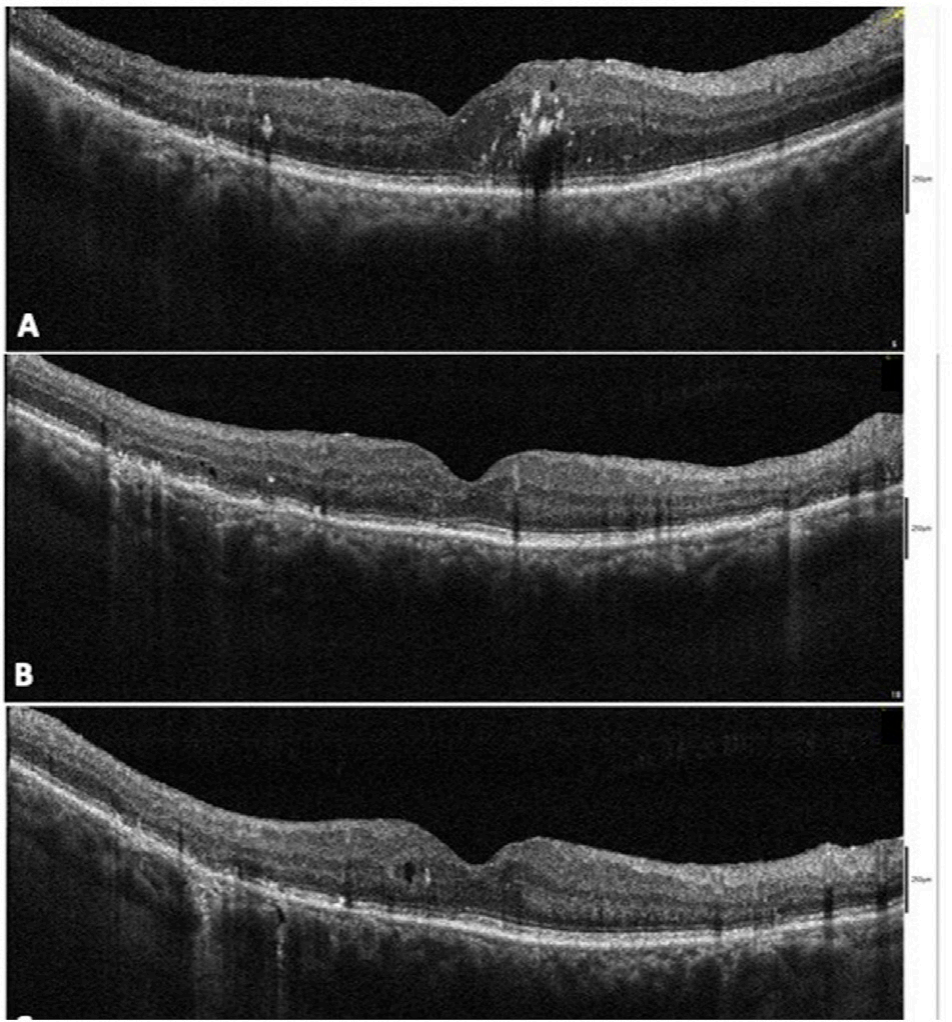
Case of persistent diabetic macular edema (DME) after pars plana vitrectomy (PPV) for tractional diabetic macular edema (TDME) treated with subthreshold micropulse yellow laser (SMYL) **(A)**. At 3 months after SMYL treatment, a reduction in the macular thickness with a normalization of the outer retinal layer was seen **(B)**. A macular thickness within the normal limits and the presence of a microcysts in the macular area at 6-month follow-up were seen **(C)**.

After SMYL treatment, the mean CMT significantly decrease from the baseline value 410.59 ± 129.91 μm to 323.50 ± 89.66 μm at 3 months (*p* < 0.001) and to 283.39 ± 73.45 μm at 6 months (*p* < 0.001) (Figure 2; Table 2).

**FIGURE 3:**
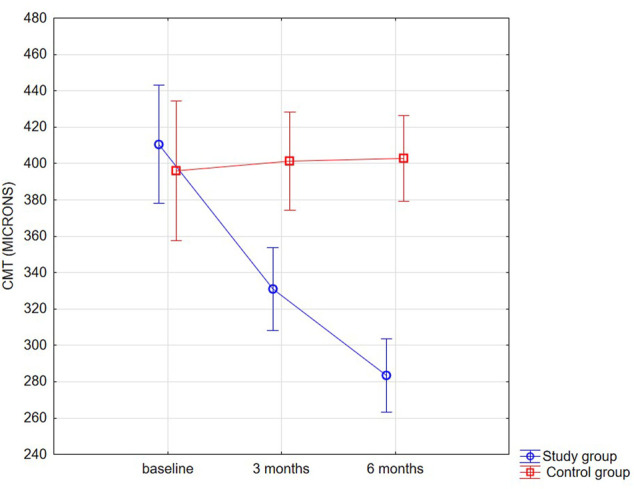
Distribution plot for CMT (*µm*) mean values in both groups at baseline and 3 and 6 months.

Additionally, in comparison to the control group, the CMT values were significantly lower in the SMYL group (interaction effect *p* < 0.001), especially at 6 months follow-up time (*p* < 0.001; Figure 3 and Table 3).

**TABLE 3:** BCVA, CMT, and OCT-A parameters of both SMYL and control groups at baseline and 3- and 6-month follow-up.

	Mean BCVA (ETDRS letters) ±SD			Mean CMT (µm) ±SD			Mean foveal VD SCP (%) ±SD			Mean foveal VD DCP (%) ±SD			Mean parafoveal VD SCP (%) ±SD			Mean parafoveal VD DCP (%) ±SD			Mean FAZ (mm^2^) ±SD		
	Baseline	3 months	6 months	Baseline	3 months	6 months	Baseline	3 months	6 months	Baseline	3 months	6 months	Baseline	3 months	6 months	Baseline	3 months	6 months	Baseline	3 months	6 months
SMYL group	51.54 ± 13.81	57.81 ± 12.82	57.83 ± 13.95	410.59 ± 129.91	331.01 ± 89.66	283.39 ± 73.45	25.51 ± 5.96	25.41 ± 5.16	27.53 ± 4.97	25.38 ± 7.15	25.76 ± 7.48	27.09 ± 4.98	42.17 ± 3.42	42.50 ± 3.75	43.43 ± 4.48	47.69 ± 3.39	48.91 ± 3.86	47.98 ± 3.44	0.29 ± 0.09	0.29 ± 0.10	0.31 ± 0.12
Control group	52.64 ± 14.64	53.81 ± 14.81	53.82 ± 15.48	396.05 ± 106.98	401.28 ± 76.36	402.84 ± 75.55	25.27 ± 6.32	24.39 ± 4.28	23.49 ± 4.08	25.34 ± 6.99	23.43 ± 4.48	24.49 ± 4.04	41.71 ± 2.41	35.44 ± 3.82	31.17 ± 3.81	47.29 ± 1.87	40.09 ± 2.78	39.94 ± 1.91	0.32 ± 0.08	0.34 ± 0.09	0.38 ± 0.09
*p*-value	1	1	1	1	0.008	<0.001	1	1	0.004	1	1	0.671	1	<0.001	<0.001	1	<0.001	<0.001	1	0.036	0.001

Abbreviations: BCVA, best-corrected visual acuity; CMT, central macular thickness; SMYL, subthreshold micropulse yellow laser; OCT-A, optical coherence tomography angiography; ETDRS, Early Treatment Diabetic Retinopathy Study; SD, standard deviation; µm, micrometers; VD, vessel density; SCP, superficial capillary plexus; DCP, deep capillary plexus; FAZ, foveal avascular zone; mm^2^, millimeter squared.

Regarding FAZ area, in SMYL Group, no statistically significant changes were seen between baseline (0.29 ± 0.09 mm^2^) and 3 (0.29 ± 0.10 mm^2^—*p* = 0.478; ANOVA) and 6 months follow-up, respectively (0.31 ± 0.12 mm^2^—*p* = 0.478; ANOVA). Similarly, no significant differences were found in the foveal and parafoveal VD in the SCP [F(2,106) = 2.973; *p* = 0.055; *η*_p_^2^ = 0.054] and DCP, respectively [F(2,106) = 0.973 *p* = 0.373; *η*_p_^2^ = 0.018]. Nevertheless, parafoveal VD in the SCP and DCP were significantly higher (interaction effect for both VD parameters *p* < 0.001) in the SMYL group when compared with the control group, respectively at 3 months (SCP *p* < 0.001; DCP *p* < 0.001) and 6 months follow-up (SCP *p* < 0.001; DCP *p* < 0.001). FAZ area was also significantly smaller in the SMYL group with respect to the control Group at 6 months follow-up (*p* = 0.001). The comparison of mean BCVA, CMT, and OCTA parameters over the follow-up course is shown in Table 3. No subjective symptoms such as visual field defects or scotoma were observed. None of the eyes experienced complications related to the SMYL treatment. A single SMYL treatment was performed in 18 eyes (33%), while 36 eyes (67%) needed a second retreatment after 3 months.

## Discussion

PPV associated with the ERM peeling is a highly effective procedure to treat patients with TDME. PPV could decrease DME through multiple mechanisms, including the release of tractional elements, improvement of intravitreal oxygenation, removal of pathological cytokines from the vitreous cavity, and acceleration of the half-life of intravitreal cytokines (Flikier et al., 2019). However, despite a functional improvement and a reduction of macular thickening, DME may not resolve completely up to 55% of treated eyes (Gunay and Erdogan, 2021) or reoccur up to 22% of patients after PPV (Pessoa et al., 2019; Fallico et al., 2021). The persistence or recurrence of macular edema can be explained by the vascular and inflammatory nature of the disease. In fact, while PPV releases the tractional component, the macular edema can be sustained by the dysfunction/breakdown of the inner and outer blood–retinal barrier (Parodi Battaglia et al., 2018).

The present prospective comparative pilot study has shown that SMYL treatment seems to be an effective and safe therapy to handle persistent DME in vitrectomized eyes following PPV for TDME leading to an improvement of both BCVA and retinal thickness after 6 months follow-up. Due to the possibility that DME may resolve slowly after PPV, SMYL therapy was deferred for up to 6 months after surgery (Behera et al., 2021), and long-term visual and anatomical outcomes in the SMYL group were compared with eyes observed after PPV without treatment (control group).

For 6 months follow-up, eyes treated with SMYL have shown both functional (from 51.54 ± 13.81 to 57.83 ± 13.95 ETDRS letters) and anatomical (from 410.59 ± 129.91 μm to 283.39 ± 73.45 μm) improvement with a single treatment in 33% and two treatments in 67% of eyes in comparison with either no visual acuity recovery either reduced macula thickness in Control group eyes.

These results suggest that in case of the persistent DME after PPV long-term follow-up did not show any significant restored visual and anatomical outcomes. Additionally, it is important to underline that SMYL is repeatable without foveal damage (Elfalah et al., 2021).

Beneficial results on the use of SMYL in the treatment of DME have been already reported by several authors in the literature (Bucolo et al., 2015; Verdina et al., 2020; Donati et al., 2021). Vujosevic et al. showed a significant improvement of BCVA from 69.7 ± 12.0 to 74.3 ± 9.5 ETDRS letters 6 months after treatment, although retinal thickness did not change during the follow-up (Vujosevic et al., 2020). This study included only naive DME eyes, which explain better visual acuity at the baseline and final follow-up.

Our results are in line with Donati et al. (Donati et al., 2021) who reported a reduction of CMT from 371.06 ± 37.8 to 326.70 ± 81.08 μm after 6 months follow-up in naïve DME eyes.

The previous authors have been already demonstrated the efficacy of micropulse laser in the treatment of macular edema in vitrectomized eyes.

Lutrull et al. (Luttrull, 2020) have reported better functional (from 0.6 ± 0.3 to 0.4 ± 0.3 logMar) and anatomical (from 364.6 ± 155.7 to 342.5 ± 112.7 μm) results after subthreshold diode micropulse laser treatment for persistent macular thickening after epiretinal membrane peeling.

In their study, the laser treatment was performed with an average of 41 months after PPV with a possible poor recovery. However, the authors stated that improvements in macular thickness and VA were not related to the natural history of progressive long-term post-membrane peeling, but to the efficacy of subthreshold diode laser macular treatment.

As regards the OCT-A parameters, our study has shown no alterations in SCP and DCP VDs in foveal and parafoveal areas, respectively, at 3 and 6 months after SMYL treatment. No modifications of the size of the FAZ were detected as well during the follow-up.

In addition, the control group eyes had significantly lower VD values at the level of the deep and superficial retinal plexi (in the parafoveal areas) and significant larger FAZ area when compared with the SMYL group. These OCT-A biomarkers confirmed progression in impaired macular perfusion in persistent DME eyes observed without treatment. It has been demonstrated that hypertension, blood pressure (BP) levels, and kidney function can affect OCTA metrics (Peng et al., 2020; Zeng et al., 2021). In the future, it would be interesting to verify the impact of hypertension, BP levels, and kidney function on OCTA metrics after SMYL treatment.

Similarly, Vujosevic et al. (Vujosevic et al., 2020) have demonstrated that micropulse laser does not alter vascular parameters such as superficial and deep VD in naïve eyes with macular edema even if they reported enlargement of the FAZ in DCP 6 months after the treatment. This could be due to a different OCT-A devise used. Indeed, the software of OCTA, used in our study, did not let to measure separately FAZ area into the SCP and DCP giving only one value measured in one slab and including superficial and deep plexi.

Currently, there are no guidelines for the treatment of persistent DME after PPV, although IVTs of anti-VEGF, corticosteroids such as DEX (Reibaldi et al., 2012; Bonfiglio et al., 2017; Fallico et al., 2021) or FA (Meireles et al., 2017; Ong et al., 2021) implants have been proved to be effective. It should be considered that the efficacy of IVTs of anti-VEGF drugs in vitrectomized eyes is significantly reduced due to the increased clearance of the drug in the vitreous cavity (Edington et al., 2017), while DEX and FA implants have shown strong anti-inflammatory activity and good efficacy due to similar clearance in vitrectomized and non-vitrectomized eyes (Edington et al., 2017; Augustin et al., 2021). However, corticosteroids implants may lead to side effects, such as an increase in IOP, cataract progression, and endophthalmitis (Vie et al., 2017), and its use is contraindicated in glaucomatous eyes (Chou et al., 2018; Celik et al., 2020). Conversely, the main advantage of the treatment with a micropulse laser is represented by its safety profile. Indeed, as already demonstrated by previous studies, it does not cause any chorioretinal foveal damage (Midena et al., 2019; Elfalah et al., 2021; Fallico et al., 2021). In our study no modifications on autofluorescence or on FFA have been detected, supporting its safety even when used on the macular area (Gawecki, 2019; Midena et al., 2021). These results confirm that SMYL therapy acts on the outer blood–retinal barrier and on the pigmented epithelium (RPE) (Gawecki 2019; Frizziero et al., 2021; Midena et al., 2021). RPE layer is considered to be the main site of action of SMYL. RPE plays an important role in the pathogenesis of DME, in outer blood–retinal barrier regulation, in homeostasis, and the integrity and survival of retinal cells. It also regulates the transport of nutrients, ions, oxygen, and water between the retina and choroid. Therefore, SMYL can reduce macular edema by acting directly on the RPE through a photostimulating effect (Gawecki, 2019). Additionally, Midena et al. (Midena et al., 2020) reported the efficacy of SMYL in the reduction of VEGF concentration and aqueous humor muller cells biomarkers in diabetic eyes.

Recently, it has been reported that subthreshold micropulse laser reduces the aqueous humor concentration of inflammatory cytokines secreted by retinal glial cells, both Müller cells, and microglial cells in eyes with DME (Midena et al., 2019; Midena et al., 2020). Inflammatory cytokines, mainly produced by the retinal microglia, were significantly reduced after treatments, suggesting that subthreshold micropulse laser may act by deactivating microglial cells, and reducing local inflammatory diabetes-related response. (Midena et al., 2019). Additionally, some authors have shown that SMYL treatment plays an anti-inflammatory role in reducing the number of HRS (a sign of activated microglia cells in the retina) (Vujosevic et al., 2020).

Recently, it has been shown that B cell activation is involved in the pathogenesis of diabetic retinopathy (Liu et al., 2020) and RPE cells can inhibit B cell activation (Sugita et al., 2010). Therefore, it could be possible that subthreshold micropulse laser could also activate RPE cells to suppress B cell activation.

The main limitations of our study are the non-randomized design and the short-term follow-up (6 months). In addition, to conclude that there is no effect on visual field sensitivity, tests such as micro perimeter should be performed.

In conclusion, this pilot study has shown the efficacy and safety of SMYL laser in vitrectomized eyes in comparison with observed eyes, suggesting its early use in the management of persistent macular edema following PPV for TDME.

A further prospective randomized study could evaluate if different anatomical features of DME, such as subretinal fluid or HRS, could have a different response to SMYL treatment.

In addition, prospective randomized studies are required to compare the efficacy of SMYL treatment with other treatment procedures, such as IVT of anti-VEGF drugs or steroids, used to treat DME in vitrectomized eyes.

## Data Availability

The raw data supporting the conclusion of this article will be made available by the authors without undue reservation.
